# Au Nanoparticle-Based Amplified DNA Detection on Poly-l-lysine Monolayer-Functionalized Electrodes

**DOI:** 10.3390/nano12020242

**Published:** 2022-01-13

**Authors:** Almudena Marti, Jurriaan Huskens

**Affiliations:** Department of Molecules & Materials, MESA+ Institute, University of Twente, P.O. Box 217, 7500 AE Enschede, The Netherlands; almudena.marti-morant@univ-lorraine.fr

**Keywords:** AuNPs, DNA sensing, signal amplification, electrochemical biosensor, poly-l-lysine

## Abstract

Affinity sensing of nucleic acids is among the most investigated areas in biosensing due to the growing importance of DNA diagnostics in healthcare research and clinical applications. Here, we report a simple electrochemical DNA detection layer, based on poly-l-lysine (PLL), in combination with gold nanoparticles (AuNPs) as a signal amplifier. The layer shows excellent reduction of non-specific binding and thereby high contrast between amplified and non-amplified signals with functionalized AuNPs; the relative change in current was 10-fold compared to the non-amplified signal. The present work may provide a general method for the detection of tumor markers based on electrochemical DNA sensing.

## 1. Introduction

Biosensors are devices that turn the specificity and selectivity of biological reactions into a detectable signal [1]. In the design of a sensor, one of the current challenges is to engineer the recognition surface in an efficient way in order to get a specific signal for the desired analyte. High control over the sensor surface allows many possibilities to optimize the sensitivity. To achieve such control, monolayers on surfaces with specific functional groups are used to control analyte selectivity at the molecular scale [2,3,4].

Among the different types of biosensors, DNA-based sensors have been arousing interest in the last decade due to their applications in many fields such as DNA diagnostics and forensics [5]. Affinity sensing of nucleic acids is among the most investigated area in biosensing due to the importance of DNA diagnosis in healthcare research and clinical applications [6]. DNA biosensors are constituted of immobilized single-strand DNA probes (ssDNA), which are able to recognize their complementary target sequences by specific hybridization, which triggers the transduction to a physical signal. This hybridization event can be measured electrochemically, optically, or gravimetrically [7]. In particular, electrochemical biosensors are promising for utilization in point-of-care diagnostics due to the fact that they are easy to use, cheap, rapid and portable [8]. 

The sensitivity and specificity of biomolecule detection is influenced by the interfacial properties of the device [9]. It has been shown that surface probe density, conformation of probe strands and density of charges on the surface influence the efficiency of nucleic acid hybridization on solid surfaces [10]. The minimization of non-specific adsorption within the sensing area is another key factor affecting the selectivity and the sensitivity of the measurement, especially when working with (ultra)low concentrations of biomolecules [11].

Various signal amplification methods exist when sensitivity needs to be improved, such as labeling probes [12], PCR [13], isothermal amplification methods [14], redox reporter molecules [15], and the use of nanomaterials [16]. There are several reports that describe the use of AuNPs for amplifying the transduction of hybridization events in electrochemical DNA sensors [17,18]. However, for the successful realization of such signals amplification strategies, proper attention to nonspecific adsorption issues that commonly control the detectability of affinity assays [17], is required. Gold nanoparticles (AuNPs) can be easily conjugated with biomolecules and retain the biochemical activity of the tagged biomolecules, making AuNPs ideal amplifiers for several biorecognition applications. In the case of the detection of nucleic acids, AuNPs can serve as carriers for the immobilization of captured probes because of their narrow size distribution, good biocompatibility, and ease of modification with thiol groups [19]. 

Here, we report the design of a surface chemistry, based on poly-l-lysine (PLL), in combination with AuNP-based signal amplification that shows excellent reduction of non-specific binding and thereby high contrast between amplified and non-amplified signals. To our knowledge, only a single paper reports the AuNP-amplified detection of DNA using a form of PLL chemistry, and only using surface plasmon resonance spectroscopy [20]. Here, we employ our earlier developed PLL methodology to introduce DNA probe moieties and antifouling oligo(ethylene glycol) (OEG) chains in a single and tunable step [11,21,22]. The detection of DNA and the integration with AuNP signal amplification is assessed using quartz crystal microbalance (QCM) and electrochemistry as benchmark analytical techniques.

## 2. Materials and Methods

### 2.1. Materials

Phosphate buffered saline tablets (PBS, pH 7.4) and sodium dodecyl sulfate (SDS) ≥ 98.5% were purchased from Sigma–Aldrich and used without further purification. The DNA sequences were purchased from Eurofins Genomics, Ebersberg, Germany (see Table S1). Milli-Q water with a resistivity >18 MΩ cm was used in all experiments. The synthesis of PLL–OEG–Mal was performed as described before [23] (Scheme S1; see also SI for a more extensive description of the synthesis procedure) and characterized using ^1^H NMR (Figure S1).

rDNA-AuNPs and nrDNA-AuNPs modification.

Gold nanoparticles (20 nm diameter) were purchased from Sigma Aldrich, Darmstadt, Germany. Reporter rDNA-modified AuNPs (rDNA-AuNPs) were prepared according to previously reported methods with slight modification [24]. Before DNA loading onto the AuNPs, the possible disulfide of thiol-modified rDNA was reduced by treatment with 100-fold excess TCEP at room temperature for 1 h. Then, 30 μL, 100 μM rDNA was mixed with 1 mL AuNPs solution and incubated at 4 °C for 12 h. After incubation, 20 μL of 1% SDS was added to stabilize the AuNPs with shaking at room temperature for 1 h. Then the rDNA-AuNPs were aged for 12 h by slowly adding 100 μL 0.5 M NaCl solution. To remove excess reagents, the solution was centrifuged at 10,000 rpm for 20 min. The wine-red rDNA-AuNPs precipitate was washed 3 times upon centrifugation with 0.01 M phosphate buffer solution (pH 7.4) and then resuspended in 0.5 mL PBS for future use. The same procedure was used to obtain the nrDNA-AuNPs.

UV-vis spectra of AuNP dispersions were recorded before and after the functionalization with the rDNA or nrDNA, as shown in Figure S2. Dynamic light scattering (DLS; Nanotrac Wave, purchased from Microtrac, via Sysmex, Etten-Leur, The Netherlands) data is shown in Figure S3. All AuNP concentrations given below are based on the concentrations of the nanoparticles (not of the DNA). Microtrac FLEX Operating software was used at 25 °C using a laser wavelength of 780 nm and a scattering angle of 90°. The observed size and standard deviation of the nanoparticles were calculated by taking averages of three measurements.

### 2.2. Methods

#### 2.2.1. Quartz Crystal Microbalance with Dissipation Monitoring (QCM-D)

Au and SiO_2_ QCM chips (AT cut, 5 MHz, 14 mm diameter) were purchased from Biolin Scientific, via Quantum Design Benelux, Grimbergen, Belgium. QCM-D measurements used a Q-Sense E4 4-channel quartz crystal microbalance with a peristaltic pump (Biolin Scientific, via Quantum Design Benelux, Grimbergen, Belgium). All experiments were performed in PBS (pH 7.4) using a flow rate of 100 μL min^−1^ at 22 °C.

Gold surfaces were cleaned for 5 min in a basic Piranha solution (Milli-Q water: H_2_O_2_ (30%): NH_4_OH (25%), in a volume ratio of 5:1:1). Thereafter, the samples were rinsed with Milli-Q water, dried in a N_2_ stream and exposed to UV-ozone (UV/Ozone ProCleaner Plus, Bioforce Nanosciences, from Nanoandmore GmbH, Wetzlar, Germany) for 20 min. 

The relationship between the measured frequency shift (Δ*f*) and the adsorbed mass per unit area (Δ*m*) was established using the Sauerbrey equation:

Δ*f* = −*C*Δ*m* (1)

where *C* is the Sauerbrey constant (17.7 ng Hz^−1^ at *f* = 5 MHz). We used the fifth overtone for the normalized frequency (Δ*f*_5_) and dissipation (Δ*D*_5_) here strictly.

#### 2.2.2. Electrochemical Measurements

Gold-on-glass sensors (200 nm gold thickness, 2.5 cm round substrates, Ssens B.V., Enschede, The Netherlands) were cleaned for 30 s using a Piranha solution (3:1 ratio of 96% H_2_SO_4_ and 30% H_2_O_2_), followed by rinsing with Milli-Q water for 20 min [23]. Thereafter, the surfaces were activated by UV–ozone for 30 min, after which they were immersed in a solution of PLL-OEG_22_-Mal_4.6_ (0.25 mg mL^−1^) in PBS (pH 7.2) for 1 h. After washing with Milli-Q water and drying in a flow of nitrogen, the chip was immersed in a solution of 1 µM of HS-DNA in PBS (pH 7.2) for 2 h. Afterwards, substrates were rinsed with Milli-Q water and dried, and then contacted by a 1 µM solution of target DNA (tDNA) in PBS at pH 7.2 for 1 h. Thereafter, the addition of the rDNA with and without AuNPs was performed. All electrolytes were freshly prepared and degassed for 15–20 min.

Cyclic voltammetry (CV), chronocoulometry (CC) and electrochemical impedance spectroscopy (EIS) were performed using PLL-modified Au substrates in a three-electrode setup (custom-built glass electrochemical cell) with a platinum disk as a counter electrode, a red rod reference electrode (Ag/AgCl, saturated KCl solution, Radiometer Analytical, via Hach Lange, Dusseldorf, Germany), and the functionalized gold substrates as working electrode (area = 0.44 cm^2^) using a 760D bipotentiostat (CH Instruments Inc., Austin, TX, USA). Data analysis was performed using CHI760D software (v. 12.04, CH Instruments Inc., Austin, TX, USA).

Chronocoulometry (CC) was used to determine the coverage of the immobilized probes on the gold surfaces. In this method, the [Ru(NH_3_)_6_]^3+^ cation (RuHex) binds to the anionic phosphodiester backbone of DNA. Under saturated conditions, assuming that the charge on the phosphate groups is entirely compensated by [Ru(NH_3_)_6_]^3+^, the amount of DNA bound at the surface can be deduced. This is performed by measuring the charge required to reduce the DNA-bound [Ru(NH_3_)_6_]^3+^ using a short reductive potential pulse. Thus, a 1000 ms pulse from an initial potential of 0.2 V to −0.5 V versus Ag/AgCl was employed to completely reduce the [Ru(NH_3_)_6_]^3+^. The concentration of the cation employed in the experiment was 50 µM to quantify the probe DNA (HS-DNA or rDNA) surface density and monitor the hybridization with the tDNA. The cyclic voltammogram (CV) was recorded after each step (see Figure S4).

When an electrode modified with DNA is placed in a low ionic strength electrolyte containing the multivalent RuHex redox cation, the RuHex becomes electrostatically trapped at that interface. The redox charge (*Q*) of RuHex can be calculated from the chronocoulometric intercept at t = 0. CC can conveniently separate the diffusion-based RuHex redox process from the surface-confined RuHex redox process, thus providing an accurate approach to measuring redox charges of RuHex confined at the electrode surface. (see SI, Quantification of DNA).

Electrochemical impedance spectroscopy (EIS).

The different steps of surface functionalization were monitored by EIS and CV. EIS measurements have been carried out in a 0.1 M K_2_SO_4_ solution containing a total concentration of 1 mM of ferri and ferrocyanide ([Fe(CN)_6_]^3−/4−^) with the following parameters: 10 kHz to 1 Hz frequency range, 5 mV AC amplitude, potential of 0.2 V. The obtained impedance spectra were analyzed by use of an equivalent circuit, which consists of a parallel circuit containing a charge transfer resistance (*R*_ct_) and a constant phase element (CPE) as well as a solution resistance (*R*_sol_) in series. The α value of the CPE was between 0.95 and 0.97, allowing evaluation of the capacitive behavior of the interfaces. The CV used a potential applied from −0.2 V to 0.6 V, at a scan rate of 100 mV/s.

## 3. Results and Discussion

**Scheme 1:**
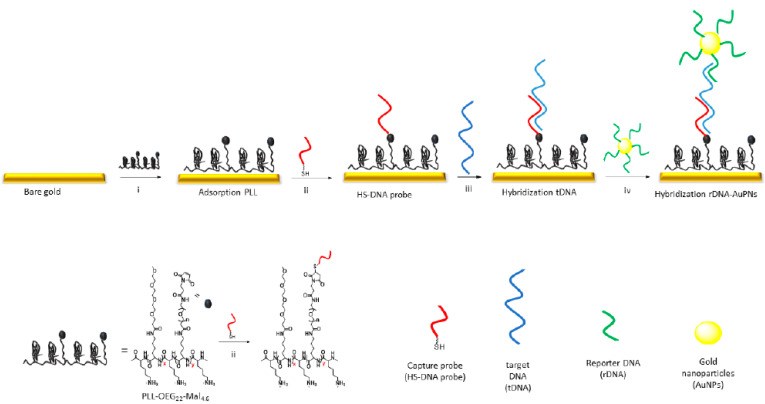
Concept of a sandwich-type DNA assay that combines PLL chemistry to provide antifouling properties and DNA probe density control with Au nanoparticle signal amplification: (**i**) self-assembly of the PLL pre-functionalized with reactive and OEG moieties, (**ii**) immobilization of capture probes (HS-DNA, red) onto a PLL-OEG-Mal-functionalized surface, (**iii**) their subsequent interaction with the target DNA (tDNA, blue) having dual recognition sites for both capture and reporter probes, and (**iv**) AuNPs tagged with reporter probes (rDNA, AuNPs, green) for hybridization to the target.

We developed a sandwich-type assay [25] in the following format (Scheme 1): self-assembly of the PLL pre-functionalized with reactive and OEG moieties, immobilization of capture probes onto the reactive groups of the PLL layer, their subsequent interaction with the target DNA having dual recognition sites for both capture and reporter probes, and finally, AuNP-tagged reporter probes that hybridize to the residual bases of the target. The positively charged PLL, grafted in a preceding single synthetic step with oligo(ethylene glycol) (OEG) groups to provide anti-fouling properties and maleimide (Mal) groups for coupling with thiol-DNA probes to yield PLL-OEG-Mal [23], was self-assembled on the electrode surface in a simple aqueous adsorption step to form a hydrophilic, reactive monolayer. A sandwich-type assay was used to show the potential application of this PLL methodology for amplified DNA detection. A thiol-DNA probe with a complementary sequence is attached to the reactive PLL monolayer, and a reporter DNA attached to AuNPs is used to amplify the signal after binding of the target DNA (tDNA). The chosen target DNA sequence belongs to GRCH38 P13, and it is a biomarker for cervical, ovarian, and gastric cancer.

Quartz crystal microbalance with dissipation monitoring (QCM-D) has been widely used in biochemical analysis as a real-time, label-free, and mass-sensitive sensing platform [22,26]. To further enhance the detection sensitivity, many efforts have been made by mass amplification such as hybridization with nanoparticles [27], in situ selective crystallization [28], biocatalyzed precipitation [29], enzymatic amplification, and so on [30]. We initially used QCM-D for detection of complementary DNA (tDNA) based on AuNP signal amplification. All DNA sequences used in this study are found in Table S1.

**Figure 1:**
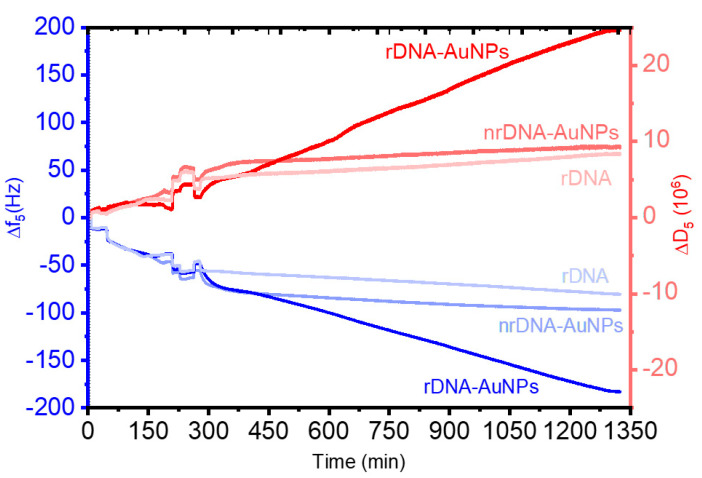
QCM-D sensograms of: the assembly of PLL-OEG_22_-Mal_4.6_ (0.25 mg/mL in PBS, pH 7.4); thiol-probe DNA (HS-DNA, 1 µM in PBS); target DNA (tDNA, 1 µM in PBS) and reporter probe DNA (rDNA, 1 µM in PBS), reporter DNA with AuNPs (rDNA-AuNPs, 0.66 nM in PBS), or non-reporter DNA (nrDNA-AuNPs, 0.7 nM in PBS) as a control experiment.

The real-time response of the surface functionalization processes, onto a Au-covered QCM substrate, of modified-PLL deposition, thiol-DNA (HS-DNA) immobilization, and consecutive hybridization with tDNA and reporter DNA (rDNA, with and without AuNPs) as well as non-complementary rDNA-functionalized AuNPs (nrDNA-AuNPs) were followed by QCM-D (Figure 1). The substrates were first activated by UV/ozone, mounted in the QCM chamber, and then a solution of PLL-OEG_22_-Mal_4.6_ (0.25 mg/mL in PBS at pH 7.4) was flushed over the sample. Upon flowing of the PLL-OEG_22_-Mal_4.6_ solution, a clear and fast frequency change (Δ*f*_5_) of ∼13 Hz was observed, which confirms the rapid adsorption of the modified polymer onto the sample surface. Upon rinsing with buffer, hardly any of the PLL desorbed, which indicates the formation of a stable monolayer of polymer). Recently, we have demonstrated that PLL functionalized with Mal allows control of the density of PNA probes on the sample surface by tuning the Mal grafting density in the preceding synthetic step [23]. This observed frequency change is in agreement with the earlier found relationship of PLL-OEG-Mal deposition versus the total degree of functionalization of the modified PLL, which indicates an expected frequency change of 13.7 Hz [23]. After the surface modification with PLL, HS-DNA (28 nt) (1 µM in PBS at pH = 7.4) was flowed over the sample surface. A clear and irreversible frequency shift was observed upon the addition of the thiol DNA, which demonstrates the stable attachment of DNA probes at the surface, as a result of the Michael-type addition reaction of the thiol probe to the Mal groups of the PLL.

Subsequently, injections of tDNA (43 nt) and rDNA (27 nt) or rDNA-AuNPs solutions produced frequency shifts (Δ*f*) of ∼13, ∼14 and ∼109 Hz, respectively. Additionally, we used nrDNA-AuNPs as a control to verify the specificity of the hybridization step, which was confirmed by an absence of signal in this case. These results demonstrate the recognition ability of the DNA interface and the feasibility of the sandwich assay, as well as the amplification of the signal using rDNA-modified AuNPs. The rather slow nanoparticle adsorption process of the specific binding rDNA-AuNPs is attributed to mass transport limitations. In later measurements, we restricted the NP adsorption time to 1 h for practical reasons.

The reported frequency shifts correspond to HS-DNA, tDNA, and rDNA densities of 6.8 × 10^12^, 2.2 × 10^12^, and 3.6 × 10^12^ molecules/cm^2^, when we assume that 80% of the mass adhering in each hybridization step arises from adsorbed water [31]. These values lead to hybridization efficiencies (while ignoring possible differences and changes in the hydration) of 35% and 160%. Different lengths of the adsorbed DNA, as well as DNA duplex formation, may cause changes in the degree of hydration, and consequently an overestimation of the hybridization efficiency, which was already observed by other groups [32,33]. 

Based on the empirical relationship between the density of PNA probes on the surface on the one hand and the Mal grafting density of PLL-OEG-Mal on the other hand, which provides 1.24 (±0.02) × 10^12^ probes per cm^2^ for each % of Mal [23], we calculated a probe density of 5.7 × 10^12^ DNA probes per cm^2^, which compares favorably to the observed density estimated for the bound HS-DNA (6.8 × 10^12^ probes per cm^2^). The difference observed between the predicted and calculated values is attributed to a difference in hydration, which cannot be deduced accurately from the QCM results.

**Figure 2:**
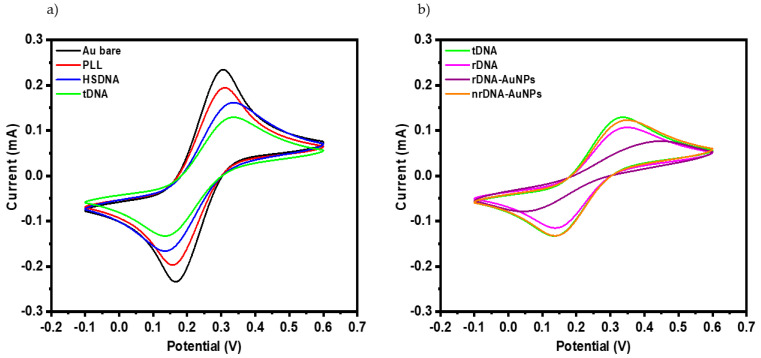
Cyclic voltammograms of gold substrates before and after coating with: monolayer of PLL-OEG_22_-Mal_4.6_ and HS-DNA, tDNA, and then followed by rDNA, rDNA-AuNPs or nrDNA-AuNPs. (**a**) Stages of preparation the sensing layer, adsorption of PLL, immobilization of the HS-DNA probe and the hybridization with the tDNA, and (**b**) studying the hybridization process between t- DNA and rDNA, rDNA-AuNPs, and nrDNA-AuNPs. All measurements were performed in 100 mM K_2_SO_4_ with 1 mM [Fe(CN)_6_]^3−/4−^ *vs* Ag/AgCl as a reference electrode (scan rate 100 mV s^−1^).

To verify the interface properties of the modified electrodes, cyclic voltammetry (CV) was performed for each step of the working electrode preparation. The gold electrodes were modified with PLL-OEG_22_-Mal_4.6_. After rinsing the sample, the electrode was immersed in a solution of HS-DNA (1 μM in PBS) for 3 h, followed by tDNA (1 μM in PBS) for 1 h (Figure 2a). Afterwards rDNA (1 μM in PBS), rDNA-AuNPs or nrDNA-AuNPs (0.6 nM) were adsorbed during 1 h (Figure 2b). CV measurements were carried out by scanning the potential from −0.2 to 0.6 V using 1 mM [Fe(CN)_6_]^3−/4−^ in 0.1 M K_2_SO_4_ as the electrolyte (Figure 2).

Figure 2 shows a reasonably fast electron-transfer kinetics of [Fe(CN)_6_]^3−/4−^ on the bare gold electrode, as indicated by a peak separation of approx. 140 mV. Adsorption of PLL-OEG_22_-Mal_4.6_ caused lower and slightly shifted oxidation and reduction peaks for the [Fe(CN)_6_]^3−/4−^ redox probe due to the presence of the PLL monolayer and the OEG grafted onto it. The difference in signal indicates the successful adsorption of the PLL-OEG_22_-Mal_4.6_ onto the surface. The signal change could be related to a blocking effect of the OEG moieties to the permeation of redox species to the surface of the electrode. The current decreased and the separation of the two peaks was enlarged after the bonding of HS-DNA, which is caused by the electrostatic repulsion between the negatively charged DNA and the Fe(CN)_6_^3−/4−^ redox species (Figure 2a). Upon the addition of tDNA and rDNA, a further decrease of the redox current and enlargement of the peak separation were observed, in line with the hybridization of both sequences. Upon hybridization with rDNA-AuNPs instead of rDNA (Figure 2b), the lowest current was obtained, indicating successful nanoparticle binding. Like in current blockade impact electrochemistry [34], we assume that the steric hindrance caused by the AuNPs contributes to the decrease of the redox current. In contrast, when using non-complementary nrDNA-modified AuNPs (nrDNA-AuNPs), the decrease of the current was minimal, which confirms the specificity of the recognition with the target DNA. All CV data indicate the successful and specific stepwise build-up of the electrochemical biosensor

**Figure 3:**
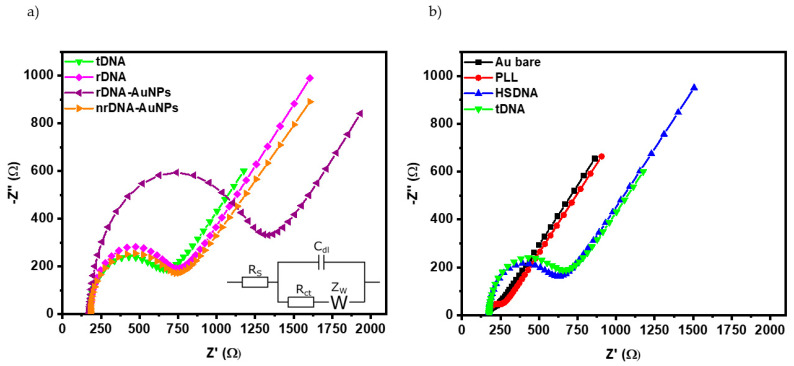
Nyquist plots of the EIS data of: (**a**) Hybridization steps of the t-DNA, followed by rDNA or the amplification step with the rDNA-AuNPs (0.66 nM), and a control experiment with non-complementary nrDNA-AuNPs (0.7 nM); (**b**) bare gold substrate before and after the subsequent adsorptions of PLL-OEG_22_-Mal_4,6_, HS-DNA probe, and 1 nM tDNA. All the experiments were performed in 100 mM K_2_SO_4_ with 1 mM [Fe(CN)_6_]^3−/4−^ at a scan rate of 100 mVs^−1^.

**Figure 4:**
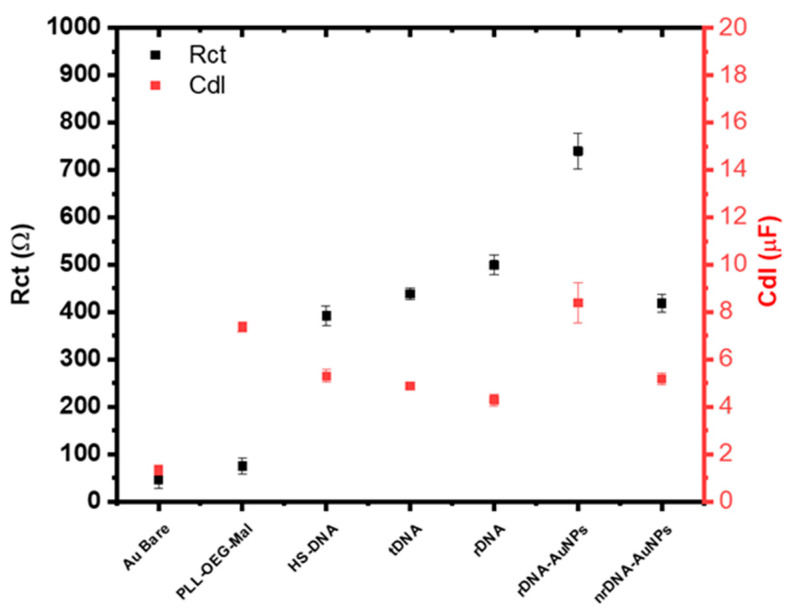
The charge transfer resistance (*R*_ct_) and double-layer capacitance (*C*_dl_) obtained by fitting the EIS data (Figure 3) to a Randles equivalent circuit (shown in the inset of Figure 3a) for each functionalization step. Standard deviations (see also Table S2) are based on three individual measurements performed on different samples, for each functionalized substrate.

Electrochemical impedance spectroscopy (EIS) was used to monitor the stepwise reaction on the surface of the modified gold electrode. EIS allows us to calculate the interfacial charge transfer resistance (*R*_ct_) between the solution and the electrode surface associated with the modification of the latter in the various steps. EIS measurements were performed in 1 mM [Fe(CN)_6_]^3−/4−^ (1:1 molar ratio) containing 0.1 M K_2_SO_4_ over a frequency range from 10 kHz to 0.1 Hz using an alternating potential with an amplitude of 5 mV, superimposed on a dc potential of 0.20 V (*vs.* Ag/AgCl). When presenting the EIS data in a Nyquist plot (Figure 3), two sections become visible. The semicircle section, at high frequencies, is related to limited electron transfer, and the charge transfer resistance (*R*_ct_) can be obtained from the diameter of the semicircle (Figure 4). The electrolyte resistance *R*_S_ can be read from the intercept with the real axis at which the semicircle ends. The other section, at low frequencies, is linear and corresponds to diffusion-limited species migration in solution, from which the Warburg impedance *Z*_W_ is determined. The double-layer capacitance (*C*_dl_) is obtained from fitting the whole curve to the Randles equivalent circuit shown in Figure 3a (inset).

The EIS data were fitted to the Randles equivalent circuit (Figure 3a, inset), and the electron transfer resistances (*R*_ct_) and double layer capacitances (*C*_dl_) were obtained (Figure 4). The data shows significant differences in the *R*_ct_ and *C*_dl_ values obtained after each surface modification step. The largest resistance value was obtained for the specific binding of the rDNA-AuNPs. In contrast, upon the addition of the non-complementary nrDNA-AuNPs, the same values as for tDNA were obtained, indicating an absence of non-specific binding. The relative changes of the resistances for the different hybridization steps were also compared using a statistical analysis (Table 1), which confirmed these conclusions. The changes observed by EIS were in agreement with those found by CV, which further demonstrated the success of the stepwise build-up of the sensor layer.

Another important issue is the representation and comparison of obtained results. In fact, due to the very high sensitivity of the EIS technique, it should be considered that the different measurements are generally performed with different electrodes or with the same unit after regeneration of the sensing surface. For that reason, results are represented as a variation of the parameter of interest (*R*_CT_) relative to the value given by the PLL immobilization to the electrode, as a blank [35]. This relative variation is represented as a ratio of delta increments, thus: Δ*s* = *R*_CT,sample_ − *R*_CT,PLL_; Δ*p* = *R*_CT,probe_ − *R*_CT,PLL_.

So, when hybridization occurred the variation ratio (∆ratio) should be >1 for the hybridization experiments and close to 1 for negative controls with non-complementary targets. Thus, the impedimetric method allows discrimination between the use of a complementary or non-complementary target for the hybridization process as we can see in Table 1. When nrDNA-AuNPs were employed, the double-strand DNA is not formed, consequently, the variation of the *R*_CT_ value was not significant, 0.94 This fact confirms that no non-specific adsorption was observed on the electrode surface.

As shown in Table 1, when the tDNA was hybridized with the HS-DNA probe, the increment of the *R*_CT_ value after the hybridization with a complementary target is attributable to the enhanced repulsion of the redox couple by the negatively charged interface, having a value of the variation of the *R*_CT_ of 1.57.

To emphasize the applicability of the combined AuNP amplification/PLL method for electrochemical biosensing, we used chronocoulometry (CC) for the detection of the adsorbed analytes on an electrode surface and the characterization of the surface coverage [36]. In CC, the surface-bound probe DNA moieties interact electrostatically with the solubilized cationic redox probe, [Ru(NH_3_)_6_]^3+^ (RuHex), which binds to the phosphate groups of the probe DNA. The CC signal correlates with the density of phosphate groups of DNA present at the surface, and a hybridization step that occurs at the surface-bound DNA probe can therefore be observed by an increase of the adsorbed RuHex redox probe [37]. By subtracting the capacitive charge (intercept at t = 0 in the absence of redox species), the total charge of surface-confined [Ru(NH_3_)_6_]^3+^ can be obtained. 

**Figure 5:**
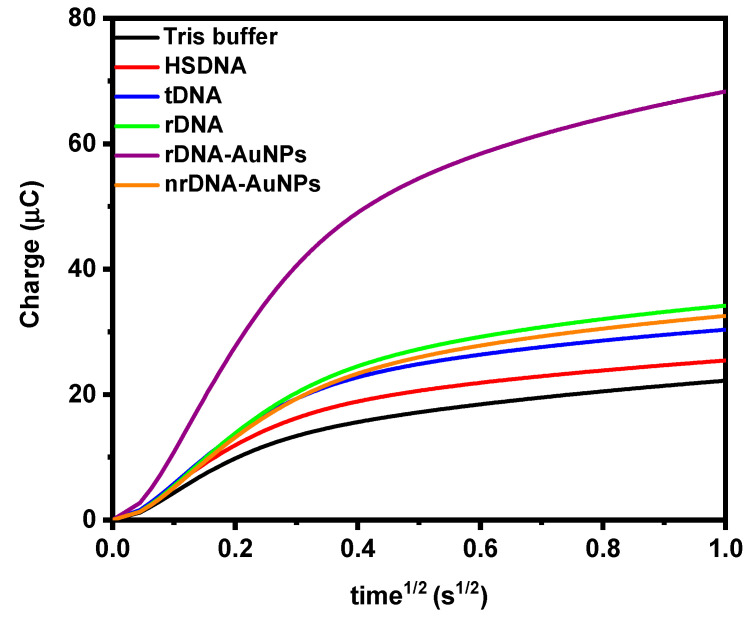
Representative chronocoulometry curves for electrodes unmodified (“Tris buffer”) or modified with HS-DNA (1 µM) in 10 mM Tris buffer (pH = 7.4) with 50 µM RuHex before and after hybridization with tDNA (1 µM), rDNA (1 µM) and rDNA-AuNPs (0.66 nM) as amplification response. Likewise, the control measurement with nrDNA-AuNPs (0.7 nM).

Here, we used the interaction of RuHex with DNA to obtain information about the hybridization of the DNA probes with target DNA at the surface, and to assess the surface coverage before and after each hybridization step. The gold electrodes were modified with PLL-OEG_22_-Mal_4.6_, and all hybridization steps were performed as described before. Typical CC curves of the gold substrates were obtained in the presence of 50 μM RuHex in 20 mM Tris buffer (Figure 5). The CVs after all functionalization steps were measured (see Figure S4). The surface density for HS-DNA resulting from the CC measurements (see Figure S5) was 7.2 × 10^12^ molecules per cm^2^. The densities of tDNA and rDNA were found to be 4.5 and 3.0 × 10^12^ molecules per cm^2^, respectively. These values indicate hybridization efficiencies of approx. 63% and 67% for the respective steps. These values agree well with results reported previously which gave densities from 1–10 × 10^12^ molecules per cm^2^ [38]. Most importantly, the values obtained after each hybridization step are very comparable to the ones calculated from the QCM data (see above). The relatively small differences can, once more, be attributed to the error in estimating the degree of hydration in the QCM measurements. Upon the binding of rDNA-AuNPs, the relative change in charge was 10-fold compared to the addition of rDNA (see calculation in SI). As observed before, a control with nrDNA-AuNPs gave hardly any change in signal, which was lower than for both rDNA and rDNA-AuNPs.

## 4. Conclusions

The results presented here show that EIS is a sensitive method to monitor changes at the interface of the electrode during the process of building the sandwich assay. Overall, the AuNP amplification in combination with PLL electrode modification shows potential for clinical applications. The electrochemical DNA sensing is based on a monolayer of PLL-OEG_22_-Mal_4.6_, for the density-controlled immobilization of probe DNA and the reduction of nonspecific binding, in combination with reporter DNA-modified AuNPs for signal amplification. The use of AuNPs increased the sensitivity markedly, while the use of the PLL enhanced the specificity. Moreover, the use of chronocoulometry allows us to have another method which, in combination with AuNPs as a signal amplifier, shows a high contrast between the amplified and non-amplified signals. The relative change in charge shows the amplification of the rDNA-AuNPs to be 10-fold compared to the signal without the AuNPs (see calculation in the SI). These electrochemical methods possess good selectivity, reproducibility, and stability.

Overall, this amplification strategy can be implemented to target different molecules by changing the DNA sequence, and thus, we envisage that this system can be used in diagnostics to detect DNA-based tumor markers, as we have recently shown in an SPR-based sensing system [20]. The implementation of AuNPs for signal amplification can lead to sensitive label-free DNA detection that can be useful in liquid biopsy analysis. This proof of concept was achieved in buffer appropriate for hybridization and should be evaluated in the future in real biological fluids such as urine, saliva, plasma, and blood.

## Figures and Tables

**Table 1:** *R*_CT_ relative variation for the sandwich experiment in hybridization assay (tDNA, rDNA, rDNA-AuNPs), non-complementary DNA assay (nrDNA-AuNPs).

Hybridization Step	∆*s*/∆*p* ± σ ^1^
tDNA	1.15 ± 0.02
rDNA	1.17 ± 0.06
rDNA-AuNPs	1.57 ± 0.08
nrDNA-AuNPs	0.94 ± 0.07

^1^ All experiments were replicated 3–4 times.

## Data Availability

Data can be obtained from the corresponding author upon personal request.

## Supplementary Materials

The following are available online at https://www.mdpi.com/article/10.3390/nano12020242/s1, Scheme S1: Synthesis of the PLL polymer, details of grafting densities of maleimide and quantification of DNA. Figure S1: ^1^H-NMR spectrum of PLL-OEG_22_-Mal_4.6_, Figure S2: UV Vis spectra of AuNPs before and after functionalization with the correspondent DNA sequence. AuNPs-citrate (commercial source) exhibits a localized peak at 524 nm, that shifts to 527 nm after functionalization with rDNA (rDNA-AuNPs) and to 526 nm after functionalization with nrDNA (nrDNA-AuNPs). Figure S3: DLS size measurements before and after the functionalization with DNA sequences. (a) citrate AuNPs, commercial source, (b) AuNPs modified with rDNA (rDNA-AuNPs) and (c) AuNPs modified with nrDNA (nrDNA-AuNPs). The size of the nanoparticles obtained by DLS were 23.1 nm, 26.3 nm and 24.9 nm respectively, Figure S4: Cyclic voltammograms of bare gold, after adsorption of PLL-OEG_22_-Mal_4.6_, after reaction with HS-DNA, after hybridization with target DNA, followed by rDNA or by AuNP amplification using rDNA-AuNPs (0.66 nM) or nrDNA-AuNPs (0.7 nM, orange line). All measurements were performed in 20 mM Tris buffer with 50 µM RuHex vs Ag/AgCl as a reference electrode (scan rate 100 mVs^−1^)., Figure S5: Representative chronocoulometry curves for gold electrodes modified with 0.25 mg/mL of PLL-OEG_22_-Mal_4.6_, before (buffer) and after reaction with 1 µM HS-DNA, and after subsequent hybridization with AuNPs-rDNA. The signal is defined as the increment of the redox charge. Q_total_ = Q_AuNPS-rDNA_-Q_dl_ or Qs_probe_- Q_dl_, Table S1: DNA sequence employed (red sequence represents capture probe-target matching bases: green sequences represent target-reporter probe matching bases). The chosen target DNA sequence belongs to GRCH38 P13, and it is a biomarker for cervical, ovarian and gastric cancer, Table S2: The standard deviation (S) and the RSD values of the EIS measurements of all the steps for the biosensing platform. The charge transfer resistance (Rct) and double layer capacitance (Cdl) were obtained by fitting the EIS data (Figure 3) to a Randles equivalent circuit for the detection of the hybridization steps between HS-DNA, tDNA and rNDA, rDNA-AuNPs and nrDNA-AuNPs. Standard deviations are based on three individual measurements performed on different samples, for each functionalized substrate. References [21,23] are cited in the supplementary materials.
