# Supplementary material for: Au Nanoparticle-Based Amplified DNA Detection on Poly-l-lysine Monolayer-Functionalized Electrodes
Source: Nanomaterials (Basel). 2022 Jan 13;12(2):242. doi: 10.3390/nano12020242 (PMC8780787; doi:10.3390/nano12020242)
Supplement: Supplementary file 1 [file nanomaterials-12-00242-s001.zip › nanomaterials-1503061-supplementary.pdf]

nanomaterials

Supporting Information

Au Nanoparticle-Based Amplified DNA Detection on Poly-L-lysine Monolayer-Functionalized Electrodes

Almudena Marti † and Jurriaan Huskens *

Department of Molecules & Materials, MESA+ Institute, University of Twente, P.O. Box 217, 7500 AE Enschede, The Netherlands; almudena.marti-morant@univ-lorraine
* Correspondence: j.huskens@utwente.nl
† Present address: Université de Lorraine, Faculté des Sciences et Technologies - Campus Aiguillettes, CNRS, L2CM, UMR 7053, Bvd des Aiguillettes, F-54506 Vandœuvre-lès-Nancy, France.

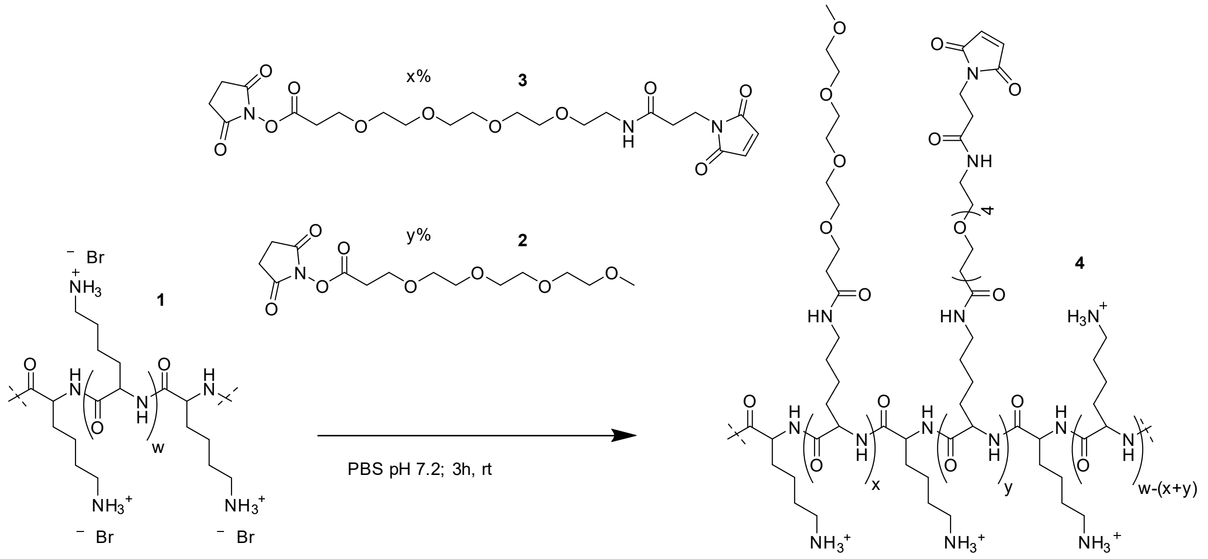

Scheme S1. Synthesis of the PLL polymer, details of grafting densities of maleimide and quantification of DNA.

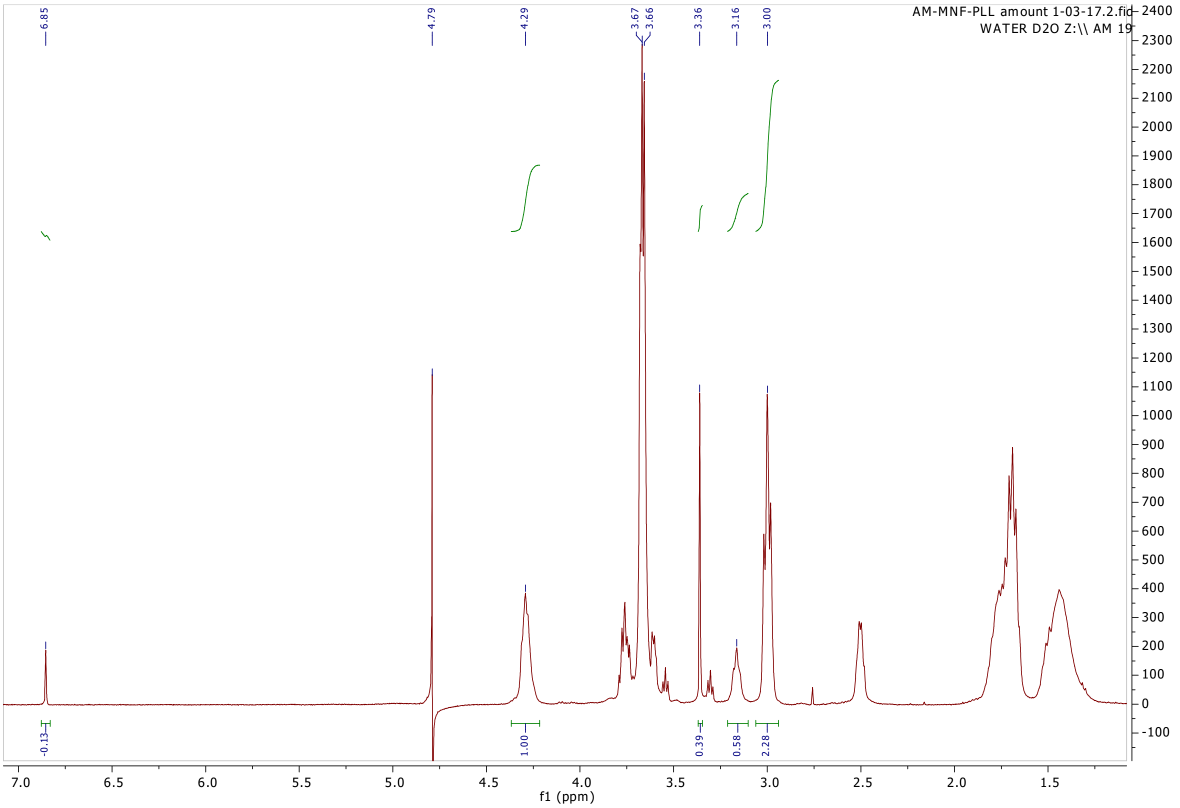

Figure S1. ^{1}H-NMR spectrum of PLL-OEG22-Mal4.6.

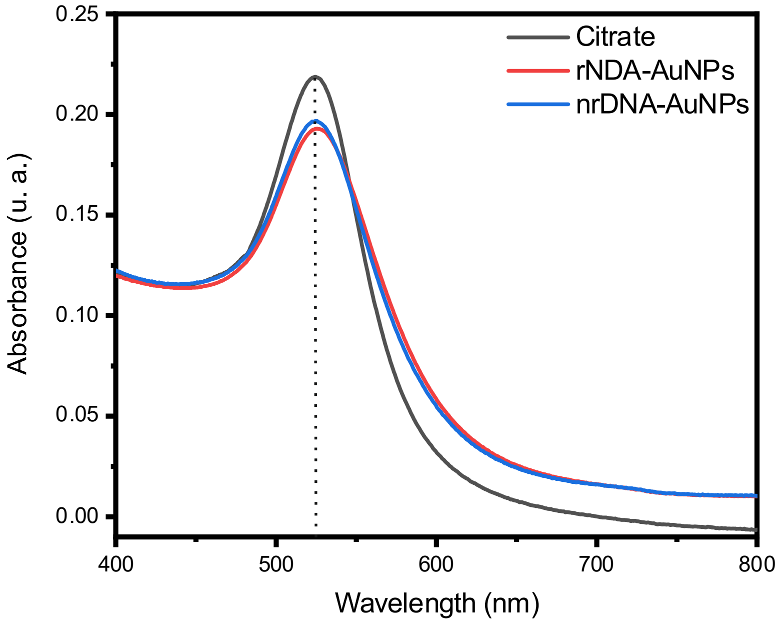

Figure S2. UV Vis spectra of AuNPs before and after functionalization with the correspondent DNA sequence. AuNPs-citrate (commercial source) exhibits a localized peak at 524 nm, that shifts to 527 nm after functionalization with rDNA (rDNA-AuNPs) and to 526 nm after functionalization with nrDNA (nrDNA-AuNPs).

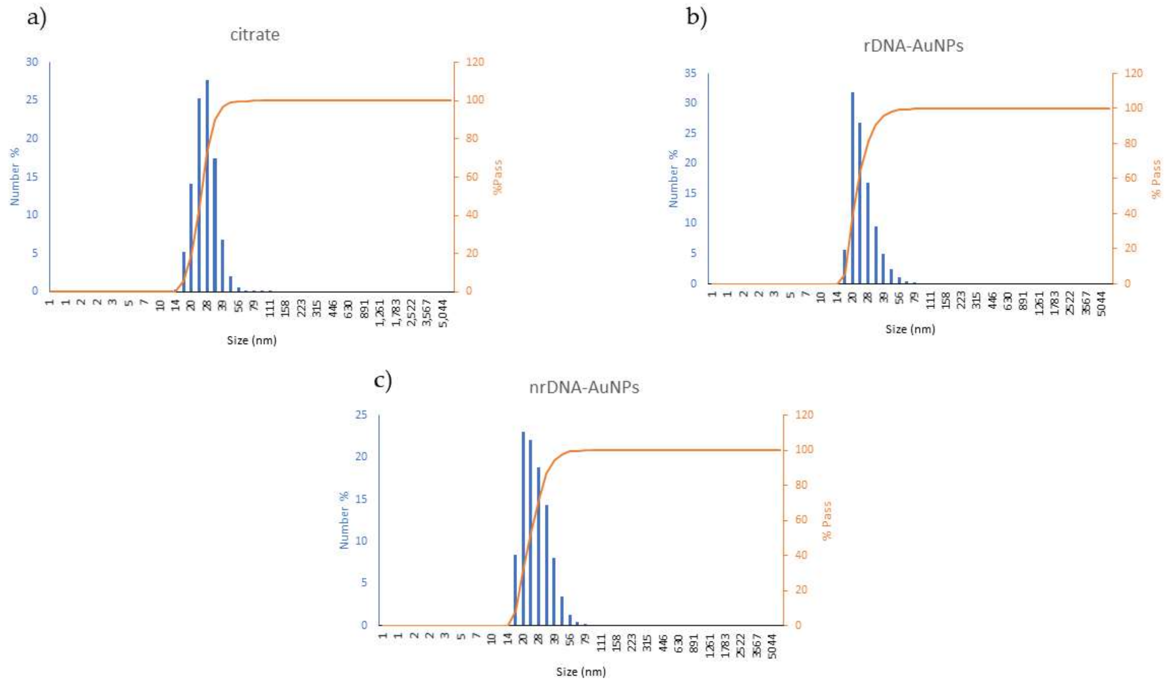

Figure S3. DLS size measurements before and after the functionalization with DNA sequences. (**a**) citrate AuNPs, commercial source, (**b**) AuNPs modified with rDNA (rDNA-AuNPs) and (**c**) AuNPs modified with nrDNA (nrDNA-AuNPs). The size of the nanoparticles obtained by DLS were 23.1 nm, 26.3 nm and 24.9 nm respectively**.**

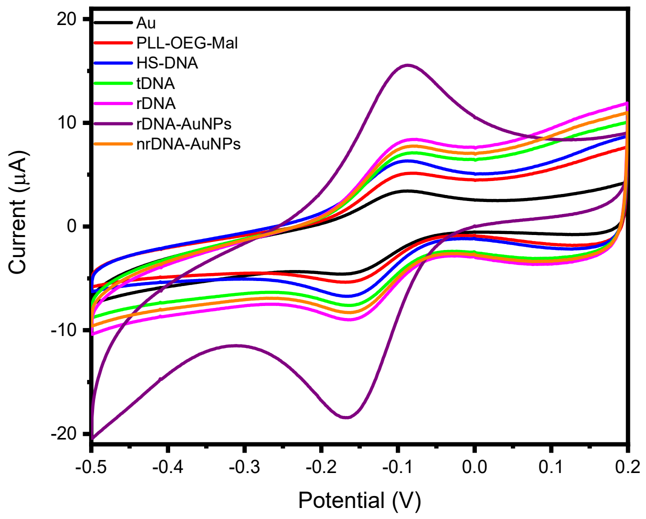

Figure S4. Cyclic voltammograms of bare gold, after adsorption of PLL-OEG$_{22}$-Mal$_{4.6}$, after reaction with HS-DNA, after hybridization with target DNA, followed by rDNA or by AuNP amplification using rDNA-AuNPs (0.66 nM) or nrDNA-AuNPs (0.7 nM, orange line). All measurements were performed in 20 mM Tris buffer with 50 μM RuHex vs Ag/AgCl as a reference electrode (scan rate 100 mVs^{-1}).

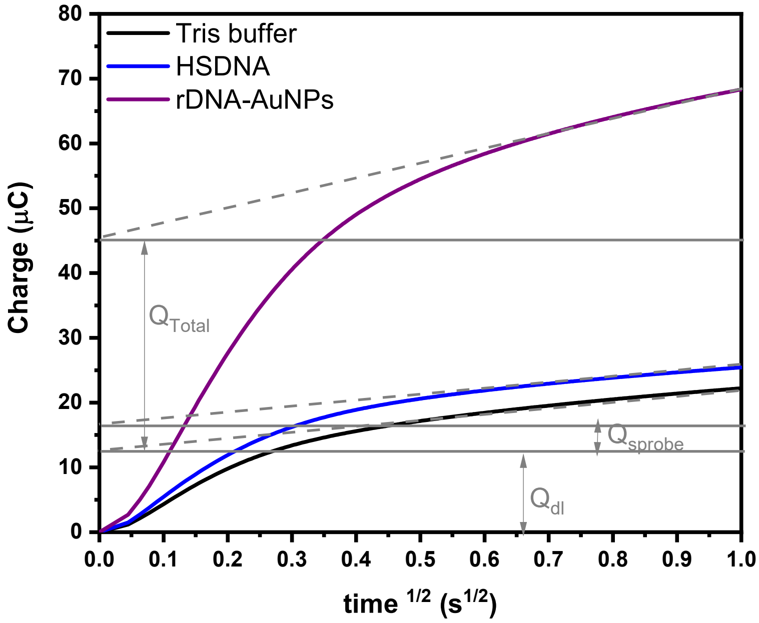

Figure S5. Representative chronocoulometry curves for gold electrodes modified with 0.25 mg/ml of PLL-OEG22-Mal4.6, before (buffer) and after reaction with 1 μM HS-DNA, and after subsequent hybridization with AuNPs-rDNA. The signal is defined as the increment of the redox charge. $Q_{total} = Q_{AuNPS\text{-}rDNA}\text{-}Q_{dl}$ or $Qs_{probe}\text{-} Q_{dl}$.

Table S1. DNA sequence employed (red sequence represents capture probe-target matching bases: green sequences represent target-reporter probe matching bases). The chosen target DNA sequence belongs to GRCH38 P13, and it is a biomarker for cervical, ovarian and gastric cancer.

Compound	Sequence (5′ to 3′)	Modification
**reporter probe (rDNA)**	GAA GGA GGG AAG GAA GGG CAA AAA AAA (27)	3′-thiol
**nrDNA**	TTT TTT TTG CCC TTC CTT CCC TCC TTC (27)	3′-thiol
**Target DNA (tDNA)**	TTG CCC TTC CTT CCC TCC TTC GTC CCC TCC TCA CAC CCC ACC C (43)	-
**Capture DNA (HS-DNA)**	AAA AAA GGG TGG GGT GTG AGG AGG GGA C (28)	5′-thiol

Table S2. The standard deviation (S) and the RSD values of the EIS measurements of all the steps for the biosensing platform. The charge transfer resistance (Rct) and double layer capacitance (Cdl) were obtained by fitting the EIS data (Figure 3) to a Randles equivalent circuit for the detection of the hybridization steps between HS-DNA, tDNA and rNDA, rDNA-AuNPs and nrDNA-AuNPs. Standard deviations are based on three individual measurements performed on different samples, for each functionalized substrate.

	Rct	Rct	RSD	Cdl	Cdl	RSD
	Ω	S	100*S/average	μF	S	100*S/average
**Au Bare**	45	16.3	36.1	1.33	0.20	15.0
**PLL-OEG-Mal**	75	17.4	23.2	7.38	0.21	2.9
**HS-DNA**	392	21.2	5.4	5.31	0.26	5.0

**tDNA**	439	12.3	2.8	4.89	0.15	3.1
**rDNA**	500	20.6	4.1	4.29	0.25	5.9
**rDNA-AuNPs**	740	37.6	5.1	8.39	0.84	10.0
**nrDNA-AuNPs**	419	19.4	4.6	5.18	0.25	4.7

Synthesis of PLL–OEG–Mal

Poly-L-lysine·HBr (PLL·HBr) (15–30 kDa), DMSO ≥99.9% anhydrous and sodium dodecyl sulfate (SDS) ≥98.5%, were purchased from Sigma-Aldrich and used without further purification. (NHS)-tetra(ethylene glycol)-maleimide (NHS-(OEG$_4$)-Mal), methyl-OEG$_4$-NHS and and Zeba™ spin desalting columns (7 kDa MWCO, 5 mL) were purchased from ThermoFischer Scientific and used without further purification. ^{1}H-NMR spectra were recorded on a Bruker 400 MHz spectrometer. Chemical shifts are reported in ppm with tetramethylsilane as an internal standard.

PLL–OEG(x)–Mal(y) with varying percentages of functionalization where synthesized based on a procedure reported previously [1] (see Scheme S1). One mL of PLL·HBr in PBS (pH 7.0), at a concentration of 10 mg mL^{-1}, was diluted with 1 mL of PBS (pH 7.4), in order to obtain a final pH of 7.2. The desired stoichiometric ratios (vs lysine monomer) of Me-OEG$_4$-NHS ester and Mal-OEG$_4$-NHS ester were added simultaneously to the mixture, under vigorous stirring, and reacted for 4 h at room temperature in a nitrogen atmosphere. The crude mixture was purified using Zeba™ spin desalting columns. For the filtration, the dialyzed solution was removed by centrifugation upon adding 2.5 mL of PBS at pH 7.0 at 1000 rpm for 2 min (twice). Then, repeated twice, 2.5 mL of the desired buffer was added and finally, the polymer solution was filtered. The treated solution was freeze-dried overnight. Final compounds were stored at −20 °C as stock solutions of 10 mg/mL in PBS 7.2. Afterwards, an NMR spectrum was recorded in D_2O with 5 μL of 0.1 M HCl using the water suppression sequence:

^{1}H NMR (400 MHz D_2O, pH 6.5) δ [ppm] = 1.26–1.55 (lysine γ-CH_2), 1.63–1.83 (lysine β, δ-CH_2), 3.00 (free lysine, H_2N-CH_2), 3.16 (OEG-maleimide, C(=O)-NH-CH_2–), 4.29 (lysine backbone, NH-CH-C(O)–), 6.86 (maleimide –C(=O)-CH-CH-C(=O)–) (see Figure S1). See below for the details of grafting densities of maleimide.

Determination of grafting densities of maleimide on PLL

The final grafting ratio (the percentage of the OEG and OEG-Maleimide chains) was determined by using the subsequent formula where the relative areas of the lysine side-chain peak (–N–CH_2) at 3.00 ppm and the OEG peak (CH_2–O–) at 3.16 ppm and the maleimide peak (C=C) at 6.86 and in ^{1}H NMR. The percentages of Tetrazine chains (y%) were calculated by the subsequent formula:

$$\%\ of\ functionalization = \frac{integral\ of\ the\ maleimide\ peak}{integral\ of\ the\ free\ lysine + integral\ of\ the\ coupled\ lysine} \times 100$$

Quantification of DNA density on modified electrodes

Calculate the surface coverage of DNA by using the following equations, detailed deduced processes were presented in the following: The total charge Q, as a function of time t is described somewhere else.[2]

$$Q_{total} = \frac{2nFAD_0^{\frac{1}{2}}C_0^{+}}{\pi^{\frac{1}{2}}} t^{\frac{1}{2}} + Q_{dl} + nFA\Gamma_0 \quad (1)$$

where n is the number of electrons in the electrode reaction (n=3), F is the Faraday constant (96485 C/equivalent), A is the electrode area (0.044cm^2), Q_{dl} is the capacitive charge (C), $nFA\Gamma_0$ is the charge produced by the adsorbed RuHex and Γ_0 is the amount of RuHex confined on the electrode surface (mol/cm^2).

The intercept at t = 0 is the sum of the capacitive charge (Q_{dl}) and the surface excess terms ($nFA\Gamma_0$).

$$Q_{total} = Q_{dl} + nFA\Gamma_0 \tag{2}$$

To achieve a much more indicative view of DNA surface density, a meaningful conversion is necessary. Γ_{DNA} is the probe coverage in molecules/cm^2. Where m is the number of base pairs in the DNA, z is the charge of the redox molecules (z=3) and N_A is Avogadro's number.

$$\Gamma_{DNA} = \Gamma_0 \left(\frac{z}{m}\right) N_A \tag{3}$$

Substituting eq 2 into eq 3:

$$\Gamma_{DNA} = \frac{Q_{total} - Q_{dl}}{nFA} \left(\frac{z}{m}\right) N_A \tag{4}$$

Upon binding of rDNA-AuNPs, the relative change in charge was 10-fold compared to the addition of rDNA, as seen in Figure 5 (main text). We use the ratio of variation of charge for each step compared to hybridization step with the tDNA:

$$\frac{\Delta Q_{rDNA-AuNPS}}{\Delta\Delta Q_{rDNA}} = \frac{Q_{rDNA-AuNPs} - Q_{tDNA}}{Q_{rDNA} - Q_{tDNA}} \tag{5}$$

At t = 1 s, the Q values for tDNA, rDNA and rDNA-AuNPs are 30.5, 33.9 and 68.5 μC, respectively, which, when filled into eq 5, leads to an 11-fold relative increase.

References

1. Movilli, J.; Rozzi, A.; Ricciardi, R.; Corradini, R.; Huskens, J. Control of Probe Density at DNA Biosensor Surfaces Using Poly(l-lysine) with Appended Reactive Groups., *Bioconjug. Chem.*, **2018**, *29*, 4110-4118.

2.. Di Iorio, D.; Marti, A.; Koeman, S.; Huskens, J. Clickable poly- l -lysine for the formation of biorecognition surfaces., *RSC Adv.*, **2019**, *9*, 35608–35613.
